# Typhoon eye effect versus ripple effect: the role of family size on mental health during the COVID-19 pandemic in Pakistan

**DOI:** 10.1186/s12992-021-00685-5

**Published:** 2021-03-29

**Authors:** Tooba Lateef, Jiyao Chen, Muhammad Tahir, Teba Abdul Lateef, Bryan Z. Chen, Jizhen Li, Stephen X. Zhang

**Affiliations:** 1 Department of Biochemistry, University of Karachi, Main University Road, Karachi, Sindh 75270 Pakistan; 2 Oregon State University, 416 Austin Hall, Corvallis, OR 97330 USA; 3 Department of Software Engineering, Sir Syed University of Engineering and Technology, ST-16, University Road, Block-5, Gulshan-e-Iqbal, Karachi, 75300 Sindh Pakistan; 4 Department of Nutrition and Dietetics, Ra’ana Liaquat Ali Khan Government College of Home Economics, Karachi, Pakistan; 5 Department of Food Science and Technology, University of Karachi, Main University Road, Karachi, Sindh 75270 Pakistan; 6 Crescent Valley High School, 4444 NW Highland Dr, Corvallis, OR 97330 USA; 7 Tsinghua University, 258A Weilun Building, Beijing, China; 8 University of Adelaide, 9-28 Nexus10 Tower, 10 Pulteney St, Adelaide, SA 5000 Australia

**Keywords:** COVID-19, Pakistan, Typhoon eye effect, Ripple effect, Family size, Mental health

## Abstract

**Background:**

The recent outbreak of COVID-19 has impacted adversely upon the mental health of millions of people worldwide. Impacts on the mental health conditions and the associated predictors relating to adults in Pakistan, the fifth most populous country in the world, during the COVID-19 remain understudied. Our aim was to investigate distress, anxiety, and overall mental health and their associated predictors among Pakistani adults in this pandemic. We specifically examine mental health issues based on the distance from the epicenter, (a predictor that has revealed opposing evidence in other countries) based on the theories of typhoon eye effect and ripple effect. The sample consisted of 601 adults who were surveyed online about 2.5 months into the outbreak across Pakistan with varying distances from the epicenter of COVID-19 of Karachi.

**Results:**

The results showed that 9.2 and 19.0% of the participants surpassed the cut-off criteria for distress and anxiety disorders, respectively. Overall, the distance from the epicenter positively predicted the mental health of adults in Pakistan, and family size negatively moderated this effect. The distance from the epicenter negatively predicted distress and anxiety disorders for adults in large families, which are quite common in Pakistan.

**Conclusion:**

The evidence of the study interestingly finds that the prediction of the mental health of people by their distance from the epicenter depends on family size. The evidence of this study can help to provide initial indicators for mental health care providers to screen vulnerable groups in Pakistan, a populous country that continues struggling to cope with the COVID-19 pandemic.

## Introduction

In Pakistan, the first case of COVID-19 appeared on February 26, 2020 in Karachi, the largest city and the financial, industrial, and trading hub of the country. The initial cases were imported to Karachi from abroad but later, community spread started, and Karachi became the initial epicenter of the virus infection [1]. As COVID-19 spread, panic among the public happened across the country, as it has happened in other countries such as Iran, Italy, Peru, and Bolivia [2–5]. For instance, one study of students has shown moderate anxiety and distress as the pandemic affected daily life activities in Pakistan [6].

It may be considered logical that people closer to the center of any disastrous event would be affected more and in turn have more mental issues whereas, the negative effects of the catastrophic event would decline for people with greater geographical distances to the epicenter. This is known as the “ripple effect” [7]. However, some findings have demonstrated an opposite and paradoxical effect referred to as “typhoon eye effect”. This was first witnessed in the 2008 Wenchuan earthquake, when it was observed that people closer to the area of crisis felt calmer [8]. Later, the same phenomenon was observed during different public health emergencies elsewhere [9–11].

In the ongoing COVID-19 pandemic, the opposing experiences of typhoon eye effect and ripple effect have been reported. Some studies have supported the ripple effect [12, 13], yet others have supported the typhoon eye effect [14–16], hence, these inconsistent findings limited the explanation of both theories. Until now, there has been no research conducted in the general population of Pakistan to assess anxiety and distress.

Therefore, the present study aimed to study mental disorders in Pakistan during the COVID-19 pandemic based on the two opposing theories of typhoon eye effect and ripple effect. Moreover, this study is the first to examine the prediction of the typhoon eye effect and ripple effect on people living within varying sizes of family, given that people tend to have larger families in countries such as Pakistan, and larger families may either drain or provide buffering resources relating to mental health issues. This study will also be one of the first medical papers to address mental health among adults in varying geographical locations in Pakistan. The findings of the research can help to pinpoint useful predictors that will help to provide targeted mental health support in vulnerable groups during the COVID-19 pandemic that continues in Pakistan, the fifth most populous country in the world.

## Methods

### Study context

The first case of COVID-19 in Pakistan was reported on February 26, 2020 in Karachi, [17], the largest city of Pakistan and the capital of Sindh, with a population of 16 million [18]. It has a high burden of disease as compared to other cities [1]. At the time of the study, February 26 to May 11, 2020, there were 9480 cases in Karachi, representing 41.5% of the 22,820 total active cases in the entire country [17]. Hence, Karachi was the clear epicenter in Pakistan at the time of the study.

### Data collection and sample

About 2.5 months into the outbreak, on May 4th – 11th 2020, we conducted an online survey of 601 adults from all over Pakistan. On May 4th, 2020, when the survey started, the total number of confirmed cases of COVID-19 in the whole country had reached 21,501, and the death toll stood at 486 [17].

The study was approved by the Institutional Bioethical Committee of the University of Karachi (IBC KU -143/2020). The participants, after their consent, filled the online survey voluntarily. The survey promised the participants confidentiality and anonymity in their responses. The participants could answer the survey in Urdu (the back-translated version) or English (the version developed originally).

### Variables

The participants reported their demographic characteristics such as age, gender, education, and marital status. They also reported their family size and daily exercise hours in the past week. We computed the distance from their geographical locations to Karachi, the COVID-19 epicenter of Pakistan.

The outcome variables included distress, anxiety, and, mental health. Distress was measured by K6, the six-item Kessler mental distress scale (0 = never, 4 = almost all of the time; α = 0.83) with the cut-off point of 13 [19, 20]. Anxiety was measured by the seven-item Generalized Anxiety Disorder-7 scale (GAD-7) (0 = never, rarely, 3 = always; α = 0.88) with the cut-off point of 10 [20–22]. Mental health was assessed by 12-item Short Form-12 (SF-12) [22–24]. SF12 cover eight sub-scales including physical functioning, physical role, body pain, general health, functionality, social functioning, emotional role, and mental health (α = 0.74).

### Data analysis approach

We used Stata 16.0 to summarize the variables and predict distress and anxiety by logistic regression and mental health by ordinary least squares regression with a 95% confidence level.

## Results

### Descriptive findings

The results showed that 47.6% of the 601 working adults were female, 62.4% were younger than 29 years old, 26.0% were between 30 to 39 years, and 11.6% were 40 years or older. 67.7% of the participants were single, 30.8% married, and 1.5% divorced. Most of the participants (70.5%) had an undergraduate degree or higher with few participants (29.0%) having a high school diploma (intermediate). On average, they exercised 0.77 h each day with an SD of 0.79 h. Overall, they had a family size of 6.03 with SD of 3.10 and resided on average 270 km away from Karachi, Sindh with SD of 510 km (Table 1).

**Table 1:** Predicting working adults’ depression disorder, anxiety disorder, and overall mental health score (*N* = 601)

Variables	n (%)	Logistic regression				Linear regression	
		Distress		Anxiety		Mental health	
		*OR* (95%CI)	*p*-value	*OR* (95%CI)	p-value	*b* (95%CI)	p-value
**Gender**							
*Male*	315 (52.4)	.74 (.42–1.33)	.315	1.09 (.72–1.65)	.688	−.98 (− 2.62–.64)	.233
*Female*	286 (47.6)						
**Age**							
*18–19*	30 (5.0)	.97 (.92–1.02)	.178	.98 (.93–1.03)	.421	.23*** (.06–.40)	.007
*20–29*	339 (57.4)						
*30–39*	156 (26.0)						
*40–79*	70 (11.6)						
**Marital status**							
*Single*	407 (67.7)	----------------------------Reference-----------------------------					
*Married*	185 (30.8)	1.09 (.46–2.59)	.845	.89 (.47–1.69)	.722	−.35 (− 2.66–1.97)	.770
*Divorced*	9 (1.7)	2.02 (.18–22.8)	.570	.69 (.08–6.45)	.748	−1.91 (− 10.70–6.86)	.668
**Education**							
*Primary*	2 (0.3)	. 95 (.51–1.76)	.865	1.10 (.67–1.80)	.698	−1.96 (−3.96–.03)	.054
*Secondary*	1 (0.2)						
*Intermediate*	175 (29.0)						
*Graduate or higher*	424 (70.5)						
**Exercise hours per day**							
*Mean [SD]*	0.77 [0.79]	.66 (.45–.96)	.028	.81 (.63–1.05)	.112	1.28 (.31–2.25)	.010
**Distance to Karachi (1000 km)**							
*Mean [SD]*	0.27 [0.51]	.54 (.18–1.6)	.265	.58 (.27–1.24)	.160	3.51 (.74–6.28)	.013
**Family size**							
*1*	5 (0.8)	.76 (.61–.94)	.013	.91 (.82–1.00)	.052	.19 (−.13–.50)	.245
*2*	17 (2.8)						
*3*	54 (9.0)						
*4*	99 (16.5)						
*5*	134 (22.3)						
*6*	122 (20.3)						
*7*	58 (9.7)						
*8*	41 (6.8)						
*9*	20 (3.3)						
*≥ 10*	51 (8.49)						
Distance * Family size		1.25 (1.04–1.49)	.017	1.14 (1.03–1.26)	.015	−.71 (−1.04 – -.38)	.000

### Descriptive and comparative findings on the outcome variables

About one-tenth of participants surpassed the cut-off criteria for distress (9.2%) and about one-fifth of participants surpassed that for anxiety (19.0%). By comparing our findings with those in 11 studies using similar measurements, we found that overall the mental health conditions of Pakistani adults were comparable or less than those in several samples in China, Spain, and Italy (Table 2 for a summary). Anxiety disorder in our sample was higher than that in a sample of adults in China in late February 2020 [27].

**Table 2:** The comparisons of adults’ distress and anxiety issues during the COVID-19 pandemic across studies

Measure	Sample description; data collection time	Prevalence	Comparison with this study	Source
**Distress**	**This study**	**9.2%**	**–**	
Kessler-6	369 adults in China, Feb 20–21, 2020	6.2%	−3.0% (−6.3 to 0.6%) *χ*^2^(1)=2.8, *p* = 0.10	[24]
Kessler-10	500 adults in Italy, April 10–13, 2020	18.6%	9.4% (5.5 to 13.3%) *χ*^2^(1)=22.2, *p* < 0.0001	[3]
Kessler-6	1599 adults in China, Feb 1–4, 2020	Mean (SD): 7.7 (±7.7)	2.2% (1.49–2.8%) T (2198) = 6.4, *p* < 0.0001	[25]
Kessler-6	2032 adults in the U.S., late April 2020	27.7%	18.5% (15.3 to 21.4%) *χ*^2^(1)=88.3, p < 0.0001	[26]
**Anxiety**	**This study**	**19.0%**	**–**	
GAD-2	3088 adults in 32 provinces of China, Feb 20–27, 2020	13.2%	−5.83% (− 2.6% to − 9.3%) *χ*^2^(1)=13.9, *p* = 0.0002	[27]
GAD-2	3480 adults in Spain, March 21–27, 2020	21.6%	2.3% (− 1.3 to 5.5%) *χ*^2^(1)=1.6, *p* = 0.21	[28]
GAD-7	103 adults in China, Feb 10–28, 2020	22.3%	3.3% (− 4.4 to 12.7%) *χ*^2^(1)=0.6, *p* = 0.44	[29]
GAD-7	98 adults in Zhongshan, Guangdong in China, Feb 15–29, 2020	23.4%	4.4% (−3.6 to 14.1%) *χ*^2^(1)=1.03, *p* = 0.31	[30]
GAD-7	4872 adults in China, Jan 31–Feb 2, 2020	22.6%	3.6% (.1–6.8%) *χ*^2^(1)=4.0, *p* = .045	[31]
GAD-2	1577 adults in Wuhan, China, Feb 18–24, 2020	23.8%	4.8% (.9–8.5%) *χ*^2^(1)=5.7, *p* = .017	[32]
GAD-7	1556 seniors older than 60 years in China	37.1%	18.1% (14.0–21.9%) *χ*^2^(1)=65.2, *p* < .0001	[33]

### Predictors of distress, anxiety, and mental health

**Fig. 1:**
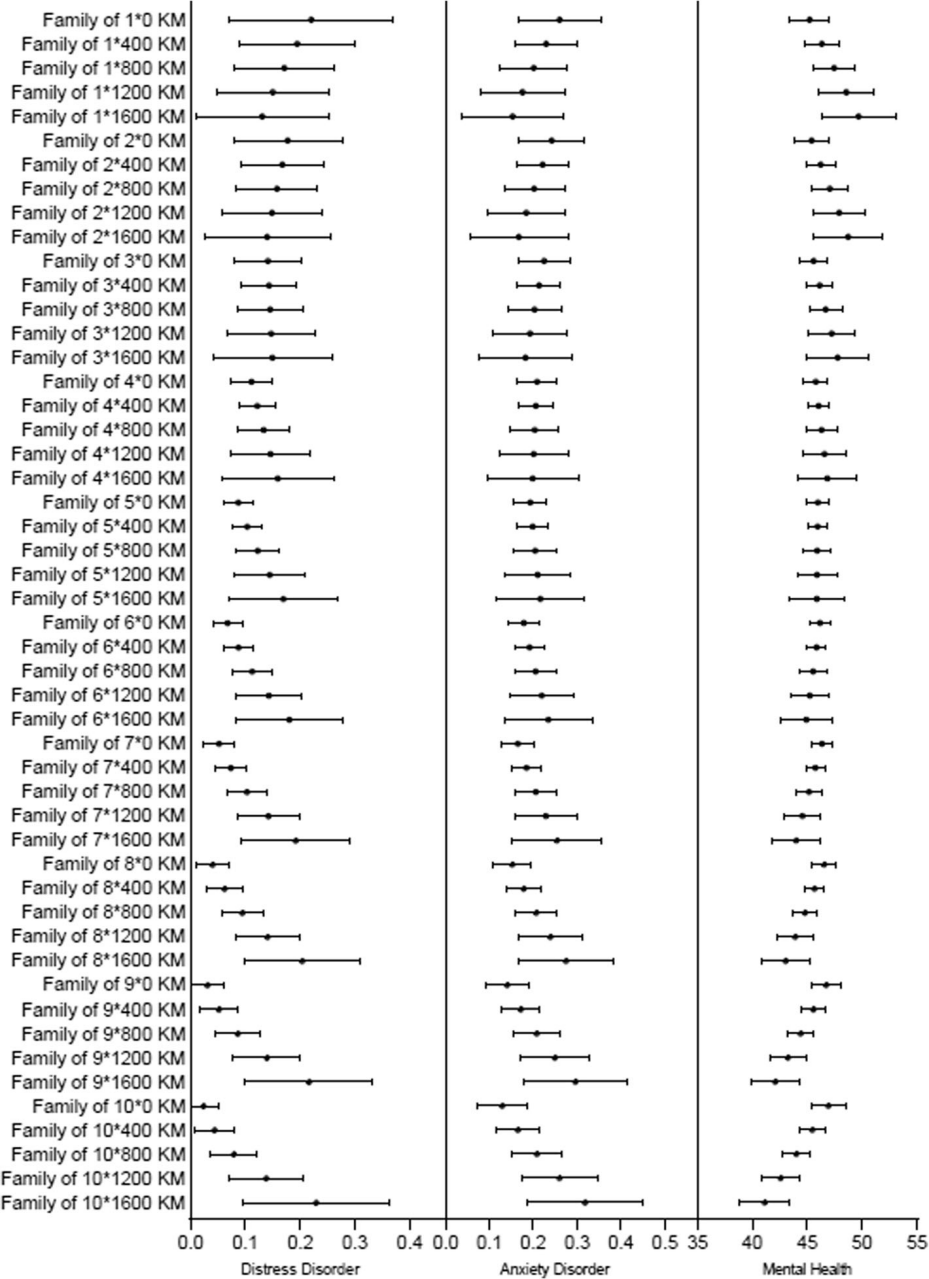
Predicted value and 95% confidence intervals of distress disorder, anxiety order and overall mental health score by family size and distance to the epicenter

The distance from the epicenter of COVID-19 in Pakistan negatively predicted the mental health of adults, but the relationship depended on their family size (b = − 0.71; 95% CI: − 1.04 to − 0.38; *P* = 0.000). Margin analysis showed that the distance from the epicenter positively predicted mental health for adults in small families (e.g. at a single-member family: b = 2.79; 95% CI: 0.28 to 5.30; *P* = 0.039). In contrast, the distance from the epicenter negatively predicted mental health for adults in large families (e.g. at an 8-member family: b = − 2.19; 95% CI: − 3.85 to − 0.54; *P* = 0.009). Similarly, the relationship of the distance from the epicenter and adults’ distress and anxiety also depended on their family size (OR = 1.25; 95% CI: 1.04 to 1.49; *P* = 0.017 for distress, and OR = 1.14; 95% CI: 1.03 to 1.26; *P* = 0.015 for anxiety). Margin analysis showed that the distance from the epicenter positively predicted distress disorder for adults in large families (e.g. for an 8-member family: OR = 0.065; 95% CI: 0.032 to 0.098; *P* = 0.000) and anxiety disorder (e.g. for an 8-member family: OR = 0.066; 95% CI: 0.008 to 0.12; *P* = 0.026) (Fig. 1).

In addition, adults who exercised more had better mental health (b = 1.28; 95% CI: 0.31 to 2.25; *P* = 0.010) and were less likely to experience distress disorders (OR = 0.66; 95% CI: 0.45 to 0.96; *P* = 0.028). The results also suggest that the older the person, the better their mental health (b = 0.28; 95% CI: 0.06 to 0.40; *P* = 0.007).

## Discussion

Pandemics have myriad impacts on the mental health of populations. In the recent outbreak of COVID-19, it has been reported that COVID-19 itself, together with many other factors has increased adverse mental health issues in various countries [5, 24, 30, 34, 35]. To the best of our knowledge, this is the first study to examine the typhoon eye effect and ripple effect at distances from the epicenter among Pakistani adults. The findings from mental distress and anxiety scales revealed the prevalence of moderate distress and anxiety in our sample. Compared to other recently published studies, the results showed that the rate of anxiety and distress among Pakistani adults was greater compared to those in China [27], but lower compared to Italy, Spain, and United States [3, 26, 28]. These differences might be due to a smaller number of reported cases and deaths in Pakistan compared to countries that have had high infection and death rates and thus greater levels of distress and anxiety. With regard to the variables associated with distress, anxiety, and mental health in Pakistan, family size and exercise were noteworthy predictors in our sample. Previous literature revealed that geographical distance from the epicenter was an important prognosticator during catastrophic events [15]. In the present study, the findings overall showed that participants residing distantly from the epicenter had better qualities of mental health with less distress and anxiety, thus supporting the ripple effect rather than the typhoon eye effect [4, 36]. However, the association could diverge based on individuals’ family size. Mental disorder decreased by the distance to the epicenter for individuals in small families, indicating the typhoon eye effect. By contrast, mental disorders increased in relation to the distance from the epicenter for individuals in larger families, showing the ripple effect.

Our results for the ripple effect versus typhoon eye effect, together with other studies on the same topic in Peru, Brazil and China [4, 16, 36], suggest the prediction of these two opposing theories may differ based on the characteristics of the countries studied. Such differences are understandable, as countries vary in their geography, media and social media reporting, medical systems, cultures, the availability of personal protective equipment (PPE), labor and employment conditions, the policies of lockdown, the ease of working from home, maintaining a living in a pandemic, and the information in both mainstream and social media [2]. The results therefore suggest the need test typhoon eye versus ripple effects as a predictive model relating to mental health in individual countries during the COVID-19 pandemic.

In our study, one of the factors that moderated and effectively reversed the manifestations of ripple versus typhoon eye effects was family size. Smaller family size was associated with less stress and anxiety, whereas larger families had higher likelihoods of distress. These findings may well be explained by heavier social or economic burdens placed on larger families confined by the lockdowns. Indeed studies have shown that financial constraints and economic hardships not only increased behavioral problems but also damaged the physical and mental health status of individuals and their families [37]. Thus, our findings identify family size as a critical contingency factor in the prediction of typhoon eye effect and ripple effect. Future research could focus on identifying unique contingency factors in individual countries, particularly in the second wave of COVID-19.

As in previous studies in Iran, Brazil and China [2, 16, 24], our sample also identified exercise hours as one of the predictors of distress, anxiety, and mental health during COVID-19. The results showed that participants who put more hours of physical activity into their daily routines had better mental health and were less likely to develop distress and anxiety symptoms. Many studies have reported that performing daily exercise can have positive impacts on anxiety and distress symptoms – see, for example, Qui et al., Peyman et al., Zhang et al., [38–40]. Due to sedentary lifestyles during the COVID-19 pandemic, it has been observed that people tended to give less attention to their physical health than in normal circumstances [41]. Thus, particularly in this pandemic era when people are extra stressed, adding physical activity to daily routines can play a role in reducing distress and anxiety. In comparison with the recent studies of Iran, China, and Brazil [39, 40, 42], age also predicted mental health in the Pakistani population. The results showed that older people had better mental health, which might be due to the extended family system in Pakistan. It has been reported that traditional extended family systems, such as those in South Asia, can contribute to healthier mental states among older people as compared to those living in smaller nuclear family systems [43]. Positive attitudes stemming from a lack of information about COVID-19 could also be another factor for better mental health of older people [44]. As compared to older people, younger people rely more on social media and the internet that have helped to spread negative information on the pandemic [14, 27]. To discern the correct information of pandemic is difficult by common people. Therefore, high usage of social media by younger people cause more panic and fear leading to poor mental health.

The overall findings of the present study can help to identify vulnerable individuals during this crisis. Exercise, family size, age, and distance from the epicenter were key predictors of distress, anxiety, and mental health in Pakistan during this pandemic, and future research could investigate their applicability to other countries. More specifically the relationship of the geographical distance to the epicenter with distress, anxiety, and mental health represented the ripple effect in large families. However, the relationship varied depending on family size and showed the typhoon eye effect in small families. Thus, the results suggest that the geographical distance from the epicenter, with an important moderating contingency of family size, can play a major role in screening of people with high risk.

This study had some limitations. During the survey dates, the total amount of active cases of COVID-19 in Pakistan had yet to reach its peak, and the situation continues to evolve. In addition, the study was conducted through an online questionnaire with the aim for a broad coverage of the adults in various parts of Pakistan, however we do not claim our sample to be representative of the adults population in Pakistan.

## Conclusion

In conclusion, the present study uncovered the prevalence of distress and anxiety disorders in a selection of Pakistani adults during COVID-19. The results indicate that geographical distance is a crucial factor in the screening of vulnerable groups and suggest the need for future studies to examine the use of the typhoon eye effect or ripple effect in terms of identifying mentally vulnerable people with a focus to identify the relevant contingency factors.

## Data Availability

The datasets presented in this article are not available. Requests to access the datasets should be directed to the corresponding author.
